# Productive Infection of Mouse Mammary Glands and Human Mammary Epithelial Cells by Zika Virus

**DOI:** 10.3390/v11100950

**Published:** 2019-10-15

**Authors:** Mathieu Hubert, Aurélie Chiche, Vincent Legros, Patricia Jeannin, Thomas Montange, Antoine Gessain, Pierre-Emmanuel Ceccaldi, Aurore Vidy

**Affiliations:** 1 Unité Épidémiologie et Physiopathologie des Virus Oncogènes, UMR Centre National de la Recherche Scientifique 3569, Département Virologie, Institut Pasteur, 75015 Paris, France; mathieu.hubert@pasteur.fr (M.H.); vincent.legros@ens-lyon.fr (V.L.); patricia.jeannin@pasteur.fr (P.J.); thomas.montange@pasteur.fr (T.M.); antoine.gessain@pasteur.fr (A.G.); 2 Université de Paris, 75013 Paris, France; 3 Groupe à 5 ans Plasticité cellulaire et Modélisation des Maladies, Département Biologie du Développement et cellules souches, Institut Pasteur, 75015 Paris, France; aurelie.chiche@pasteur.fr

**Keywords:** Zika virus, dissemination, mammary glands, tropism, primary cells, luminal cells, myoepithelial cells

## Abstract

Zika virus (ZIKV) belongs to the large category of arboviruses. Surprisingly, several human-to-human transmissions of ZIKV have been notified, either following sexual intercourse or from the mother to fetus during pregnancy. Importantly, high viral loads have been detected in the human breast milk of infected mothers, and the existence of breastfeeding as a new mode of mother-to-child transmission of ZIKV was recently hypothesized. However, the maternal origin of infectious particles in breast milk is currently unknown. Here, we show that ZIKV disseminates to the mammary glands of infected mice after both systemic and local exposure with differential kinetics. Ex vivo, we demonstrate that primary human mammary epithelial cells were sensitive and permissive to ZIKV infection in this study. Moreover, by using in vitro models, we prove that mammary luminal- and myoepithelial-phenotype cell lines are both able to produce important virus progeny after ZIKV exposure. Our data suggest that the dissemination of ZIKV to the mammary glands and subsequent infection of the mammary epithelium could be one mechanism of viral excretion in human breast milk.

## 1. Introduction

Zika virus (ZIKV) is a positive single-stranded RNA virus and belongs to the *Flavivirus* genus in the *Flaviviridae* family. As part of the large category of arthropod-borne viruses, or arboviruses, ZIKV is mainly transmitted to humans by mosquito bites, especially from infected *Aedes aegypti* and/or *Aedes albopictus* [1]. However, during the last emergence in Latin America (2015–2016), several non-vector-borne transmissions were notified, such as horizontally following sexual intercourse [2,3] and pseudo-vertically by a transplacental route [4]. Interestingly, viral genome and infectious particles were detected in genital secretions (semen [5,6] and vaginal secretions [7,8]) and amniotic fluid [9], but also in numerous other human body fluids, such as urine [10,11,12,13], saliva [14,15], tears [16], nasopharyngeal swabs [17], and breast milk [15,18,19,20,21,22]. Altogether, these bodily fluids could represent an efficient vehicle for the human-to-human transmission of ZIKV.

In particular, four arguments strengthen the plausibility of breastfeeding as a risk for the mother-to-child transmission of ZIKV. First, the breast milk of lactating ZIKV-infected mothers has been shown to harbor a high viral burden (2,9.10^4^ to 2,4.10^6^ viral RNA copies/mL), and the presence of infectious particles has been confirmed in both the colostrum and mature breast milk [19,22]. Second, the experimental susceptibility of rhesus and cynomolgus macaques to ZIKV infection after oropharyngeal and intra-gastric inoculations suggests that ZIKV can be orally transmitted. Third, two studies have demonstrated that fresh breast milk does not exert any short-term antiviral activity [23,24]. Fourth, evidence of the mother-to-child transmission of ZIKV via breast milk was recently highlighted in a 5-year-old child [25,26].

Actually, cellular and molecular mechanisms underlying mother-to-child transmissions of ZIKV via breastfeeding have been poorly studied. In particular, the maternal origin of infectious particles in breast milk remains unknown, but the detection of viral genome in breast milk over 30 days after the onset of illness [22], when viremia is null, suggests the existence of a potential viral niche in the mammary gland.

The viral excretion of ZIKV in breast milk requires the transfer of infectious entities from the blood to the milk compartment. The blood–milk barrier is formed by the mammary epithelium, which is bistratified and composed of two main cell types [27]: luminal cells, which form an inner layer and produce/secrete milk during the lactation phase, and basal myoepithelial cells, which form an outer layer and contract alveola to eject milk to the nipple. A single study has explored the relationship between ZIKV and the mammary glands [28]. In their study, Regla et al. demonstrated that the systemic infection of lactating AG129 mice, which lack both types I and II interferon (IFN) receptors, led to infection of myoepithelial and immune cells of the mammary glands and viral excretion in breast milk [28].

Here, we demonstrated that local or natural-mimicking infection also leads to ZIKV dissemination to the mammary glands. By using the A129 mouse model, which lacks only the type I IFN receptor, we compared the dissemination process of ZIKV to the mammary glands of systemically- and locally-infected mice and observed differential kinetics of viral dissemination according to the administration route. *In vitro*, we showed that primary human mammary epithelial cells were able to release important viral particles in the extracellular medium, and that both luminal and myoepithelial human cell lines were permissive to ZIKV infection, suggesting that infection of the mammary epithelium could be an important key feature for viral excretion in breast milk.

## 2. Materials and Methods

### 2.1. Cell Culture

Normal human primary mammary epithelial cells (HMEpiC, ScienCell Research Laboratories, Carlsbad, CA, USA) were cultured in Mammary Epithelial Cell Medium (MEpiCM, ScienCell Research Laboratories) supplemented with Mammary Epithelial Cell Growth Supplement (MEpiCG, ScienCell Research Laboratories) and penicillin/streptomycin (ScienCell Research Laboratories) onto poly-L-lysine-coated plates (ScienCell Research Laboratories). Luminal human mammary epithelial cells (MCF-7 cell line, ATCC HTB-22) were cultivated in Dulbecco’s Modified Eagle Medium (DMEM/F12) supplemented with L-glutamine (Gibco, Life Technologies, Carlsbad, CA, USA), 10% fetal bovine serum (FBS), 100 U/mL penicillin, 100 μg/mL streptomycin, 20 ng/mL human epidermal growth factor (hEGF, Peprotech, Neuilly-Sur-Seine, France), 0,5 μg/mL hydrocortisone (Sigma, St. Louis, MO, USA), and 10 μg/mL insulin (Sigma). Myoepithelial human mammary epithelial cells (MDA-MB-231 cell line, ATCC HTB-26) were grown in DMEM supplemented with L-glutamine (Gibco), 10% fetal bovine serum (FBS, Gibco), 100 U/mL penicillin, and 100 μg/mL streptomycin (Gibco). Vero cells (ATCC CRL-1586) were grown in DMEM supplemented with L-glutamine (Gibco), 10% fetal bovine serum (FBS, Gibco), 100 U/mL penicillin, and 100 μg/mL streptomycin (Gibco).

### 2.2. Mice

A129 mice (IFNAR^−/−^ [29]) were housed and bred in the Institut Pasteur animal facilities accredited by the French Ministry of Agriculture for breeding and performing experiments on live rodents.

### 2.3. Virus Strains

Human isolates of ZIKV belonging to the African lineage (strain HD78788, GenBank: KF383039) and the Asian lineage (strain H/PF13, GenBank: KX369547; strain Brazil/2016, GenBank: KU991811) were kindly provided by V. Choumet (Institut Pasteur, Paris, France) and amplified through a limited number of passages on mammalian cells (Vero cells). Briefly, ZIKV was allowed to adsorb to 90% confluent Vero E6 cell monolayers for 2 h at 37 °C, 5% CO_2_, in DMEM supplemented with 2% FBS. After incubation, inoculum was replaced by DMEM–2% FBS until cytopathic effects appeared (3–4 days of infection). Finally, supernatants were centrifuged and frozen at −80 °C until titration. All viral titers were determined by a foci forming assay (FFA) on Vero E6 cells.

### 2.4. Foci Forming Assay

Samples were serial diluted in DMEM–2% FBS and applied onto 90% confluence Vero cell monolayers for 2 h at 37 °C, 5% CO2. After viral adsorption, inocula were removed and replaced by a mixed solution of DMEM–4% FBS and PBS–1% carboxymethyl cellulose in a 1:1 ratio for 3 days. After 3 days, cells were fixated in PBS–4% paraformaldehyde for 15 min at room temperature (RT), and permeabilized with PBS–0.1% Triton for 3 min at RT. Non-specific sites were blocked with PBS–1% bovine serum albumin–0.1% Tween 20 for 30 min at RT. ZIKV envelope protein (E) staining was performed using the mouse anti-E (clone 4G2) antibody as the primary antibody and the horseradish peroxidase (HRP)-conjugated goat anti-mouse IgG antibody (Biorad, Hercules, CA, USA) as the secondary antibody. Finally, freshly prepared peroxidase substrate (Vector Vip; Vector Laboratories) was added for 5–15 min and the foci of infection were manually counted.

### 2.5. Animal Infections

Eight to 14-week-old A129 female mice were intraperitoneally or subcutaneously infected with 3.2.10^5^ to 6.6.10^6^ foci forming units (FFU) of the American (Brazil/2016) strain of ZIKV. Infection was monitored in the plasma, spleen, inguinal lymph nodes (iLN), and iLN-depleted mammary glands. Before tissue harvesting, mice were perfused with PBS, organs were weighed, and the viral burden was measured by qRT-PCR. Mice were killed when the weight loss of ≥20% or ≥10% in association with one suffering symptom was observed.

### 2.6. Cell Infections

Cells were infected with the African (HD78788), Asian (H/PF13), or American (Brazil/2016) strains of ZIKV at different multiplicities of infection (MOI), as indicated in each experiment. After 2 to 4 h of adsorption, cells were washed in PBS and medium was replaced by 2% FBS–DMEM. At several time points, infection was monitored by immunofluorescence and quantified by flow cytometry after immunostaining of the ZIKV envelope protein (E), as described below. Viral production in the supernatant was analyzed by qRT-PCR and a foci forming assay.

### 2.7. Immunofluorescence

To monitor ZIKV infection in cells, supernatant was removed at the indicated times and cells were rinsed in PBS. Cells were fixed in PBS–4% paraformaldehyde (Electron Microscopy Sciences, Hatfield, PA, USA) for 15 min at room temperature (RT), and permeabilized with PBS–0.1% Triton (Sigma) for 3 min at RT. Non-specific sites were blocked with PBS–1% bovine serum albumin (BSA, Sigma)–0.1% Tween 20 (Sigma) for 30 min at RT. ZIKV envelope protein (E) staining was performed using a primary mouse anti-flaviviral E antibody (4G2; home-purified from the ATCC hybridoma [30]) diluted in PBS–0.2% BSA–0.2% Tween 20 for 1 h at RT, and a secondary Alexa Fluor 488-coupled goat anti-mouse antibody (Life technologies) diluted in PBS–0.2% BSA for 30 min at RT. Finally, cells were mounted in Fluoromount G–DAPI (SouthernBiotech, Birmingham, AL, USA) and imaged on a fluorescence microscope (EVOS FL, Life Technologies). Cells were washed twice with PBS between each step.

### 2.8. Flow Cytometry

To quantify ZIKV-infected cells, supernatant was removed at 48 h post-infection and cells were rinsed in PBS. Dissociation of cells was performed by trypsinization (Gibco, by Life technologies). Intracellular staining of E was performed as for immunofluorescence, except that antibodies were incubated for 20 to 40 min at RT. Finally, data were acquired by fluorescence-activated cell sorting (FACS) using Gallios and CytoFLEX Beckman Coulter cytometers, and analysis was performed using FlowJo 10.0.8r1 software.

### 2.9. Ethics Statement

To quantify ZIKV viral production, cell supernatants were harvested at different times post-infection and centrifuged to remove cellular debris, and viral RNA was extracted (QIAamp Viral RNA Mini Kit, Qiagen), following the manufacturer’s recommendations. Neonatal mice organs and mothers’ mammary gland RNA extractions were performed using Trizol (Ambion, by Life Technologies). Other mothers’ organ RNA extractions were performed using the RNeasy Plus Mini Kit (Qiagen). Reverse transcription was performed in a 20 μL final volume from 11 μL of template RNA using random hexamers and the SuperScript IV First-Strand cDNA Synthesis or Maxima H Minus Reverse-Transcriptase kits (Invitrogen, Carlsbad, CA, USA). Quantitative PCR was performed in a 20 μL final volume from 5 uL of template cDNA, supplemented with10 μL of MasterMix (iTaq Universal SYBR Green Supermix, Biorad), 500 nM of each ZIKV NS5-specific primer (Forward: 5′–AAR TAC ACA TAC CAR AAC AAA GTG GT–3′; Reverse: 5′–TCC RCT CCC YCT YTG GTC TTG–3′), and using the following program: 10 min/95 °C, followed by 40 three-step cycles of 15 s/95 °C, 20 s/60 °C, and 30 s/72 °C (Mastercycler Eppendorf Realplex, Hamburg, Germany). Quantification analysis was performed using a standard curve of a ZIKV-encoding plasmid.

### 2.10. Ethics Statement

Anonymized normal human primary mammary epithelial cells (HMEpiC) were obtained in compliance with local, state, and federal laws and regulations governing the procurement and distribution of human tissue, and provided by ScienCell Research Laboratories (Carlsbad, CA, USA). Work on animals was performed in compliance with French and European regulations on the care and protection of laboratory animals (EC Directive 2010/63, French Law 2013-118, February 6th, 2013). All experiments were approved by the Ethics Committee #89 and registered by the French “Ministère de l’Enseignement supérieur, de la Recherche et de l’Innovation” under the reference “APAFIS#9594-2017041412342250v3” (date of approval: 11/06/2018). The usage of genetically modified mice (A129) was approved by the institutional instances and the French “Ministère de l’Enseignement supérieur, de la Recherche et de l’Innovation” under the reference n 2194 (date of approval: 06/10/2017).

### 2.11. Statistical Analysis

All statistical analyses were performed using Prism 7 software. All data were representative of three independent experiments except when mentioned in the legend, and presented as the mean ± SD. Each test was detailed in figure legends.

## 3. Results

### 3.1. ZIKV Spreads to the Mammary Glands of Systemically- and Locally-Infected A129 Mice with Differential Kinetics

**Figure 1:**
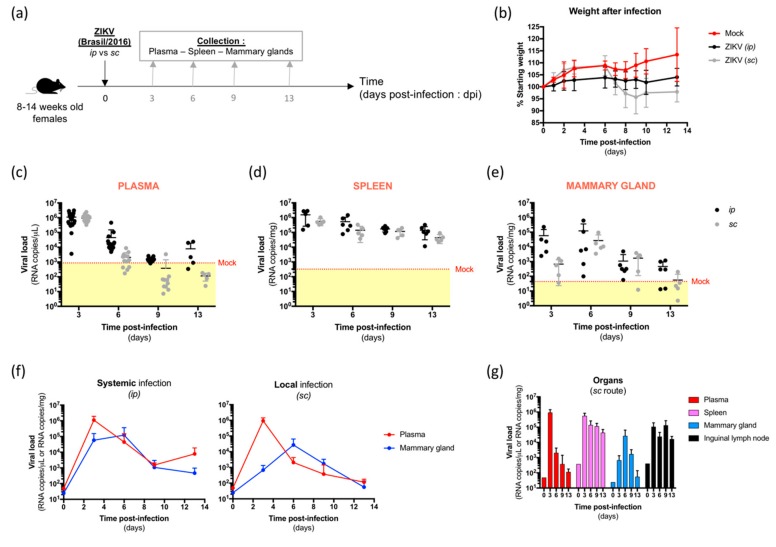
Kinetics of dissemination of the Zika virus (ZIKV) to the mammary glands of systemically- and locally-infected mice. (**a**) 8–14-week-old immunodeficient mice (A129) were systemically (*ip*, 3,2.10^6^ FFU) or locally (*sc*, 6,6.10^6^ FFU) infected by ZIKV (Brazil/2016). Plasma, spleen, and inguinal lymph node (iLN)-depleted mammary glands were collected at 3, 6, 9, and 13 days post-infection (dpi) for viral load quantification. (**b**) Body weight evolution of mice was monitored and results are expressed as a percentage of the starting weight. (**c**–**e**). The viral burden was measured in the plasma (**b**), spleen (**c**), and mammary gland (**d**) by SYBR Green-based qRT-PCR using NS5-specific primers and expressed as the mean ± SD of ZIKV RNA copies/µL of plasma or mg of tissue. The dotted line shows the specificity limit of the method, which was evaluated from mock-treated mice, and represents the threshold under which values were considered as “not detected”. (**f**) Representation of the viral load evolution in the bloodstream (red) and the mammary glands (blue) of systemically- (*ip,* left panel) and locally- (*sc,* right panel) infected mice. Results are expressed as ZIKV RNA copies/μL of plasma or mg of tissue. (**g**) Recapitulative histograms of the viral burden in organs of locally infected mice. Results are expressed as ZIKV RNA copies/μL of plasma or mg of tissue.

Several case studies reported the detection of ZIKV genome or infectious particles in the breast milk of infected mothers [15,19,21,22,26]. Infectious particles were also detected in the mammary glands and breast milk of systemically infected AG129 mice [28]. However, whether ZIKV is able to spread to the mammary glands of infected women after a mosquito bite remains unknown. Therefore, we used a well-established mouse model of ZIKV infection and pathogenesis [31] to explore and compare dissemination of the virus in the mammary glands after systemic and local infections. First, we inoculated 8–14-week-old A129 female mice via the intra-peritoneal route with the American (Brazil/2016) strain of ZIKV and monitored body weight loss and the ZIKV dissemination profile over time (Figure 1a). We noted that intraperitoneally ZIKV-exposed mice did not gain weight compared to mock-treated mice (Figure 1b, black line), and developed viraemia at 3 days post-infection (dpi), which declined at 6 dpi (Figure 1c, black symbols), confirming a typical acute infection of ZIKV-exposed mice. As expected, we confirmed that ZIKV was present in the spleen as soon as 3 dpi and persisted at 6, 9, and 13 dpi when viraemia declined (Figure 1d, black symbols). Interestingly, ZIKV was found in the mammary glands of intraperitoneally-infected mice as early as 3 and 6 dpi, before declining at 9 and 13 dpi (Figure 1e, black symbols), suggesting a rapid dissemination of ZIKV from the bloodstream to the mammary glands.

Then, because ZIKV is mainly transmitted via mosquito bites, we locally infected 8–14-week-old A129 female mice via the subcutaneous route with the same strain of ZIKV (Figure 1a) to confirm the viral dissemination to mammary glands and compare its kinetics. ZIKV-exposed mice lost weight from 6 dpi without any other associated suffering symptoms (Figure 1b, grey line), and developed a peak of viraemia whose intensity and kinetics are similar to systemically-infected mice (Figure 1c, grey symbols). As for systemic infection, ZIKV was detected in the spleen from 3 to 13 days after local infection (Figure 1d, grey symbols). However, we detected a delayed dissemination profile of ZIKV to the mammary gland in locally- compared to systemically-infected mice (Figure 1e, grey symbols). Indeed, the peak of viral load in the mammary glands became apparent at 6 dpi after subcutaneous infection (Figure 1f, right panel) in contrast to 3 dpi after intraperitoneal infection (Figure 1f, left panel). Then, mammary viral loads decreased over time after both systemic and local infection, confirming that the virus does not persist in this organ. Importantly, ablation of the inguinal lymph node (iLN) before measurement of the viral burden in the mammary gland permitted us to avoid viral RNA contamination, particularly rich in infected target cells (Figure 1g). Taken together, these results demonstrate that ZIKV is able to spread to the mammary glands of both systemically- and locally-infected mice, with delayed kinetics after local infection.

### 3.2. ZIKV Productively Infects Human Primary Mammary Epithelial Cells

**Figure 2:**
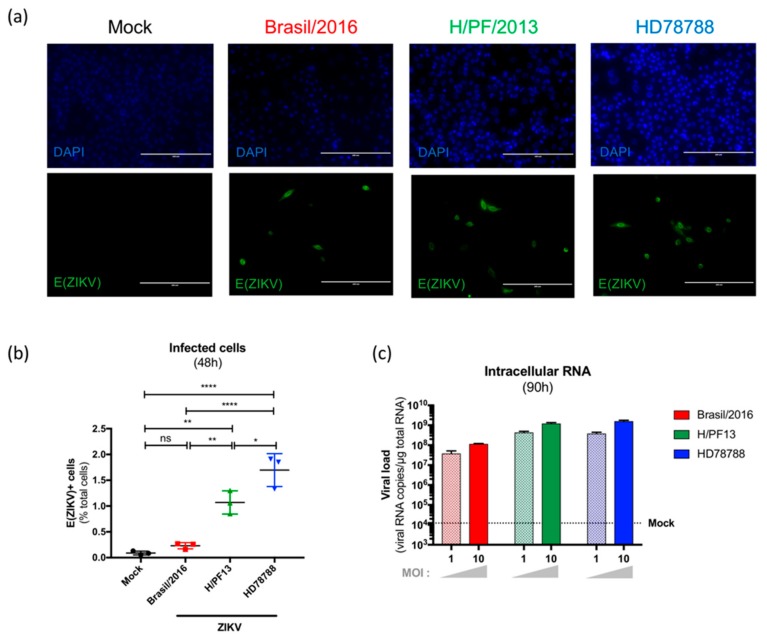
Infection of primary human mammary epithelial cells (hMECs) by ZIKV. Primary hMECs were exposed to three strains of ZIKV (Brazil/2016, H/PF13, and HD78788) at multiplicities of infection (MOI) 1 and 10 for 48 h. (**a**) At 48 h post-infection (MOI 10), the envelope protein of ZIKV (E(ZIKV)) expression was visualized by immunofluorescence using a pan-*Flavivirus* antibody (4G2; green). Nuclei were stained with DAPI (blue). Bar scale: 200 µm. (**b**) E(ZIKV)-positive cells were quantified by flow cytometry in hMECSs exposed or not exposed to ZIKV at MOI 1. Statistical test: ordinary one-way ANOVA. * *p* < 0.05; ** *p* < 0.005; *** *p* < 0.0005; **** *p* < 0.0001. (**c**) At 90h post-infection, intracellular RNA was extracted, and the ZIKV NS5 expression level was quantified by SYBR Green-based qRT-PCR. Results are expressed as viral RNA copies/µg total RNA. The dotted line shows the specificity limit of the method, which was evaluated from mock-treated hMECs, and represents the threshold under which values were considered as “not detected”. Results are representative of two independent experiments.

As we demonstrated the presence of ZIKV in mammary glands of subcutaneously-infected mice, we wondered whether mammary epithelial cells could be a support for ZIKV replication and play a role in ZIKV excretion in breast milk. To test this hypothesis, we exposed human primary mammary epithelial cells (hMECs) isolated from a healthy human breast tissue to three different strains of ZIKV (MOI 10): an African-lineage strain (HD78788), an Asian-lineage strain (H/PF13), and an Asian-lineage strain isolated during the Latin America outbreak (Brazil/2016). No destruction of the cell monolayer was observed upon ZIKV exposure (Figure 2a).

As a characteristic of *Flavivirus* infection, we noted a typical perinuclear staining of ZIKV E envelope protein (E(ZIKV)) in some ZIKV-exposed primary hMECs, but not in mock-treated cells (Figure 2a). Then, we selected and quantified infected cells harboring a strong E(ZIKV) expression by flow cytometry and showed that hMECs were susceptible to all tested ZIKV strains (Figure 2b). As described in the literature for other cell type infections, the African strain (HD78788) shows a higher infection rate of hMEC (1.70 ± 0.32% of infected cells) than the Asian (H/PF13) and American (Brazil/2016) strains (1.07 ± 0.23% and 0.23 ± 0.06% of infected cells, respectively) (Figure 2b). Moreover, compared to these rather low percentages of infected cells, high intracellular viral RNA levels were detected in all ZIKV-exposed hMECs at 90h post-infection (for both MOI 1 and 10), confirming the active replication of ZIKV in those cells (Figure 2c).

**Figure 3:**
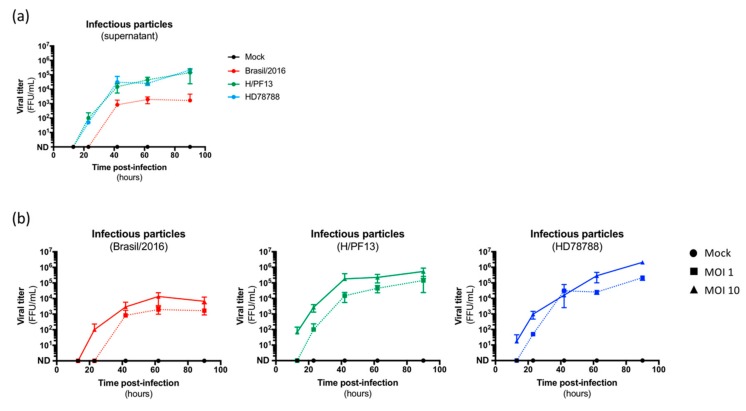
Production and release of infectious particles by ZIKV-infected hMECs. (**a**) Viral production of infectious particles in the supernatant was measured over time by a foci forming assay on Vero cells. (**b**) Dose-dependent production of ZIKV infectious particles by hMECS exposed or not exposed (Mock, black) to three strains of ZIKV (Brazil/2016, red; H/PF13, green; HD78788, blue).

Finally, we wondered whether this limited infection led to the production of infectious particles in the supernatant. As shown in Figure 3a, the number of infectious particles in the supernatant of ZIKV-exposed hMECs strongly increased over time (Figure 3a, colored curves) compared to mock-treated cells (Figure 3a, black curve), demonstrating that this infection efficiently produced viral progeny. Interestingly, higher viral loads were measured in the supernatant of H/PF13- and HD788-infected cells compared to Brazil/2016-infected cells (Figure 3a), in agreement with the number of infected cells and the intracellular viral burden. Additionally, the viral production of infectious particles seems to be dose-dependent (Figure 3b).

Altogether, these data prove that the mammary epithelial cell constitutes an efficient system to produce and release ZIKV infectious particles, despite a limited infection rate, and could be implicated in viral excretion in breast milk.

### 3.3. Both Human Luminal and Myoepithelial Cells of the Mammary Epithelium are Permissive to ZIKV Infection

**Figure 4:**
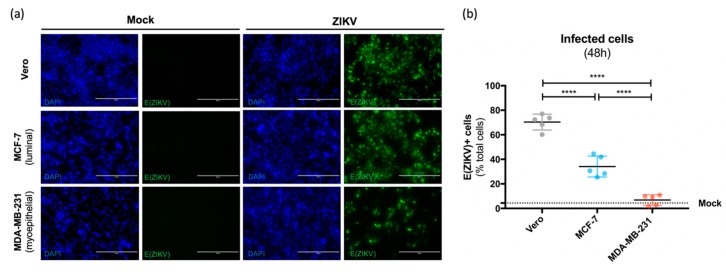
Infection of luminal and myoepithelial cell lines from the human mammary epithelium by ZIKV. Luminal (MCF-7) and myoepithelial (MDA-MB-231) human mammary epithelial cell lines were exposed to ZIKV (Brazil/2016) at MOI 1. Vero cells were used as a positive control for ZIKV infection. (**a**) At 48 h post-infection, the envelope protein of ZIKV (E(ZIKV)) expression was visualized by immunofluorescence using a pan-*Flavivirus* antibody (4G2; green). Nuclei were stained with DAPI (blue). Bar scale: 200 µm. (**b**) ZIKV-infected cells were quantified by flow cytometry examining E(ZIKV) expression at 48 h post-infection. Statistical test: ordinary one way ANOVA. * *p* < 0.05; ** *p* < 0.005; *** *p* < 0.0005; **** *p* < 0.0001.

As we showed that ZIKV productively infects human primary mammary epithelial cells, we wondered whether a specific cell type could be targeted. To address this issue, we assessed the permissivity of luminal-phenotype (MCF-7) and myoepithelial-phenotype (MDA-MB-231) human cell lines to ZIKV infection. After exposing MCF-7 and MDA-MB-231 cells to the American (Brazil/2016) strain of ZIKV at MOI 1 for 48 h, we observed a typical perinuclear staining of ZIKV envelope protein, E(ZIKV), in both cell types (Figure 4a). No staining was detected in mock-treated cells.

Moreover, the quantification of E(ZIKV)^+^ cells by flow cytometry revealed a differential infection rate according to the luminal or myoepithelial phenotype. Indeed, only 7% of myoepithelial (MDA-MB-231) cells showed a strong expression of E(ZIKV) at 48 h post-infection (Figure 4b), while about 35% of luminal (MCF-7) cells were positive for E(ZIKV). As a positive control, 70% of the highly permissive Vero cells were infected.

**Figure 5:**
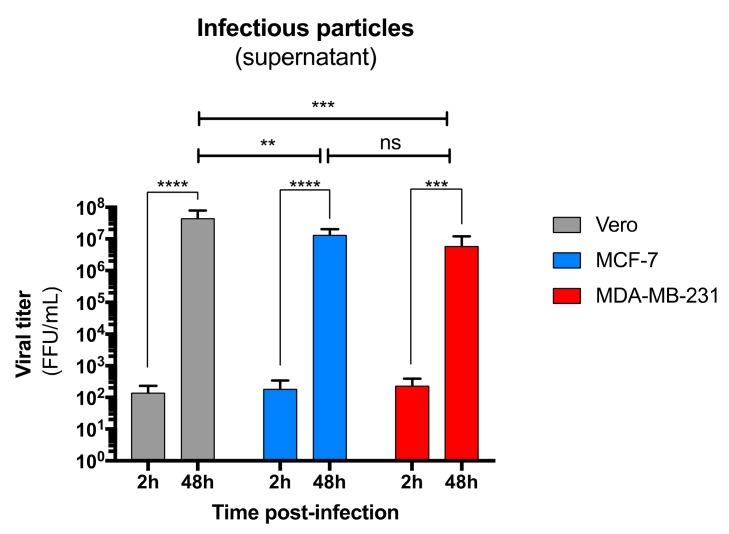
Production and release of infectious particles by ZIKV-infected luminal and myoepithelial cells. Viral production of infectious particles in the supernatant was measured over time by a foci forming assay on Vero cells. Statistical test: one-way ANOVA coupled to a ratio paired t-test. * *p* < 0.05; ** *p* < 0.005; *** *p* < 0.0005; **** *p* < 0.0001.

Next, we wondered whether the infection of both mammary epithelial cell types could contribute to producing viral progeny in the supernatant. As shown in Figure 5, the number of infectious particles in the supernatant of MCF-7 and MDA-MB-231 cells strongly increased over time, demonstrating that both infected MCF-7 and MDA-MB-231 cells efficiently produced viral progeny (Figure 5). Interestingly, viral titers in the supernatant of both MCF-7 and MDA-MB-231 cells were similar to those of the supernatant of Vero cells, confirming the efficiency of the mammary epithelial cell system in producing ZIKV infectious particles. Luminal MCF-7 and myoepithelial MDA-MB-231 cells were also susceptible to both African and Asian lineages of ZIKV [32].

Taken together, these results demonstrate that ZIKV productively infects both luminal and myoepithelial cells of the mammary epithelium, which could play a mutualist role in ZIKV excretion in breast milk.

## 4. Discussion

During the recent outbreaks, ZIKV was detected in the breast milk of several infected mothers [4,15,19,20,21,22,25,26]. Although the nature of the viral entity remains unknown—either cell-free or cell-associated—breast milk was shown to be infectious [19,21,22,26]. Several mechanisms could explain the infectivity of breast milk: productive infection of the blood–milk barrier and transmigration of circulating infected cells. However, high viral loads of ZIKV were detected in the breast milk 33 days after the onset of symptoms and in the absence of any viremia [22], strengthening the first hypothesis and suggesting a potential reservoir role of the mammary gland. Interestingly, the isolation of infectious particles from the colostrum and the mature breast milk [22], when the mammary epithelium is impermeable, suggests an active mechanism of viral translocation from the bloodstream to the breast milk. In this study, we used an in vivo model of ZIKV infection to explore the viral dissemination of ZIKV to the mammary glands and we performed in vitro experiments to investigate the cell tropism of ZIKV in the mammary epithelium.

Because non-structural protein 5 (NS5) of ZIKV is not able to inhibit the murine IFN response pathway [33], immunocompetent mice are not susceptible to ZIKV infection. Therefore, several animal models have been developed, which are mostly deficient in the IFN pathway, such as A129 (deficient for the IFN-type I receptor) and AG129 (deficient for both the IFN-type I and II receptors). In the context of an in vivo infection, it is well-described that ZIKV disseminates in numerous tissues of infected immunocompromised mice such as those of the spleen, liver, kidney, testes, brain, and spinal cord [31], but whether the virus is present in the mammary gland remained unknown for a long time. Recently, one study provided the demonstration of ZIKV dissemination and replication in lactating mammary glands of AG129 mice after systemic infection [28]. In our study, we confirmed that ZIKV was also able to rapidly spread to non-lactating mammary glands following a systemic infection in less immunocompromised mice (A129). Since we limited the blood-derived ZIKV contamination of mammary glands by perfusing mice with PBS before sampling and separated the inguinal lymph nodes from mammary glands, which are rich in ZIKV target cells, we can conclude that the mammary glands of mice are targeted by ZIKV during acute infection. Moreover, we found that ZIKV also spread to the mammary glands after a more natural-mimicking local infection. Notably, contrary to in the brain, we showed that the dissemination of ZIKV to the mammary glands looks like a peak, with differential kinetics according to the infection route: whereas ZIKV viral load peaks in mammary glands as early as 3 dpi after systemic infection (intraperitoneal), the peak is delayed to 6 dpi after local infection (subcutaneous). However, by using AG129 mice, Regla-Nava et al. demonstrated that ZIKV was present in lactating mammary glands at 5 dpi and persisted at the same level until at least 11 dpi. Indeed, mammary glands are dynamic organs which undergo important architectural and metabolic changes according to their status (puberty, nulliparous, gestation, lactation, and involution) [27], and ZIKV dissemination, replication, and clearance can differ, depending on the mammary gland status. Therefore, ZIKV could persist longer in lactating than non-lactating mammary glands. Two main hypotheses could explain the differences in viral clearance from the lactating vs. non-lactating mammary gland. First, mammary epithelial cells are more abundant in lactating glands [27] and could act as an active reservoir for permanent viral excretion in breast milk. Second, differentiation-induced phenotypic changes occurring during lactation could allow epithelial cells to be more sensitive to ZIKV infection. The mammary epithelium serves as a frontier between the bloodstream and the milk compartment. It shapes the lactiferous ducts/alveoli walls, and is bilayered. The inner layer is composed of luminal cells, which produce and secrete milk in the lumen during the lactation phase, and the outer layer is composed of myoepithelial cells, which contract alveoli to eject milk to the nipple. By using human cell lines, we found that ZIKV was able to infect both luminal and myoepithelial mammary epithelial cells, inducing important viral progeny production. Indeed, a few infected cells was sufficient to release comparable amounts of infectious particles to highly permissive Vero cells, demonstrating the high ability of these cells to produce ZIKV virions. These results complete the *in vivo* detection of ZIKV NS2B expression in murine myoepithelial cells after systemic infection of AG129 mice by Regla-Nava et al. Moreover, as human mammary epithelial cell lines may not fully recapitulate the function of epithelial cells found *in vivo*, we also showed that primary human mammary epithelial cells can be productively infected by ZIKV, strengthening their potential role in ZIKV excretion in breast milk. In the lactation phase, luminal cells undergo a differentiation process, resulting in specialized secretory cells which produce and secret milk in alveola [34]. As ZIKV exits the cell by exocytosis, exploiting the secretion machinery of milk-producing luminal cells could be optimal for the virus to be excreted. Moreover, apocrine secretion could also be hijacked to release vesicle-cloaked viral clusters in extracellular vesicles. The fact that basal myoepithelial cells are also permissive to ZIKV infection let us hypothesize viral reaching of the mammary gland by successive infections of myoepithelial and luminal cells. However, we cannot exclude that ZIKV-infected immune cells could also be exploited as a Trojan horse during their transmigration from the bloodstream to the breast milk. Nonetheless, viral genome was detected in the breast milk of a lactating woman 33 days after the onset of symptoms in the absence of any viremia [22], suggesting the existence of a long-term blood-independent origin of ZIKV in breast milk. Therefore, we also propose a second hypothesis which combines a first reaching of ZIKV to the mammary glands via the transmigration of infected immune cells at the moment the patient is viremic, followed by a de novo infection of proliferating mammary epithelial cells in the alveolar compartment, which could act as a reservoir in the mammary gland as long as the lactation state is maintained.

## 5. Conclusions

As a conclusion, we demonstrated that ZIKV was able to disseminate to the mammary glands of A129 mice after both systemic and local exposure, suggesting that the vector-borne transmission of ZIKV via mosquito bites could result in mammary gland infection. Interestingly, we revealed that the viral dissemination kinetics to the mammary glands is dependent of the administration route and is delayed after local exposure compared to systemic exposure. In the mammary glands, the blood–milk barrier is composed of a bilayered epithelium of luminal and myoepithelial cells. In vitro, we showed that both cell types were permissive to ZIKV infection, as well as human primary mammary epithelial cells, suggesting a potential excretion of ZIKV in breast milk by productive infection of the mammary epithelium. This study is the first to explore the kinetics of ZIKV dissemination to the mammary glands after a local and natural-mimicking administration route and to demonstrate the permissivity of human mammary epithelial cells to ZIKV infection. If the mother-to-child transmission of ZIKV via breastfeeding is experimentally demonstrated, this work could serve as a basis for other studies to better understand the molecular mechanisms of viral excretion in breast milk and the implementation of prevention strategies.
